# Associations of lifetime walking and weight bearing exercise with accelerometer-measured high impact physical activity in later life

**DOI:** 10.1016/j.pmedr.2017.10.011

**Published:** 2017-10-25

**Authors:** Ahmed Elhakeem, Kimberly Hannam, Kevin C. Deere, April Hartley, Emma M. Clark, Charlotte Moss, Mark H. Edwards, Elaine Dennison, Tim Gaysin, Diana Kuh, Andrew Wong, Kenneth R. Fox, Cyrus Cooper, Rachel Cooper, Jon H. Tobias

**Affiliations:** a Musculoskeletal Research Unit, Translational Health Sciences, Bristol Medical School, University of Bristol, UK; b MRC Lifecourse Epidemiology Unit, University of Southampton, UK; c MRC Unit for Lifelong Health and Ageing at UCL, UK; d Centre for Exercise Nutrition and Health Sciences, University of Bristol, UK

**Keywords:** Accelerometer, Exercise, Life course, Physical activity

## Abstract

High impact physical activity (PA) is thought to benefit bone. We examined associations of lifetime walking and weight bearing exercise with accelerometer-measured high impact and overall PA in later life. Data were from 848 participants (66.2% female, mean age = 72.4 years) from the Cohort for Skeletal Health in Bristol and Avon, Hertfordshire Cohort Study and MRC National Survey of Health and Development. Acceleration peaks from seven-day hip-worn accelerometer recordings were used to derive counts of high impact and overall PA. Walking and weight bearing exercise up to age 18, between 18–29, 30–49 and since age 50 were recalled using questionnaires. Responses in each age category were dichotomised and cumulative scores derived. Linear regression was used for analysis. Greater lifetime walking was related to higher overall, but not high impact PA, whereas greater lifetime weight bearing exercise was related to higher overall and high impact PA. For example, fully-adjusted differences in log-overall and log-high impact PA respectively for highest versus lowest lifetime scores were: walking [0.224 (0.087, 0.362) and 0.239 (− 0.058, 0.536)], and weight bearing exercise [0.754 (0.432, 1.076) and 0.587 (0.270, 0.904)]. For both walking and weight bearing exercise, associations were strongest in the ‘since age 50’ category. Those reporting the most walking and weight bearing exercise since age 50 had highest overall and high impact PA, e.g. fully-adjusted difference in log-high impact PA versus least walking and weight bearing exercise = 0.588 (0.226, 0.951). Promoting walking and weight bearing exercise from midlife may help increase potentially osteogenic PA levels in later life.

## Highlights

• Exposure to higher impact PA in old age is thought to improve skeletal health.
• It is unclear how walking and exercise history relate to high impact PA in old age.
• More frequent weight bearing exercise was more strongly related to high impact PA.
• Associations were strongest for highest levels of walking and weight bearing exercise.
• Performing both activity types from midlife might promote osteogenic PA at old age.

## 1 Introduction

The many health benefits of physical activity (PA) include reduced risk of chronic diseases (Lee et al., 2012), increases in bone mineral density (Marques et al., 2012), prevention of falls and fractures (Gardner et al., 2000, Qu et al., 2014) and maintenance of physical capability (Cooper et al., 2011) and independent living during older age (McPhee et al., 2016). It is thought that benefits of PA for bone are mediated by deformations caused by higher impacts or loading forces, leading to new bone which subsequently reduces risk of osteoporosis (Hannam et al., 2016a, Martyn-St James and Carroll, 2009, Tobias et al., 2014). For example, we recently developed (Deere et al., 2016) an accelerometer-based method for characterising PA according to vertical impact, and showed that positive associations between PA and lower limb bone strength in postmenopausal women were explained by exposure to vertical impacts ≥ 1.5 g, despite their rarity (Hannam et al., 2016a). To develop interventions that are more effective at promoting higher impact PA in later life (and subsequent accrual of associated benefits for bone), a better understanding of the determinants of higher impact PA is required.

Prior history of PA has been identified as an important correlate of current PA (Bauman et al., 2012) and it is thought that PA is relatively stable across life though tracking becomes weaker as time between measures increases (Hirvensalo and Lintunen, 2011, Telama, 2009). For example, in the Medical Research Council (MRC) National Survey of Health and Development (NSHD), greater leisure-time PA at ages 36, 43 and 53 were related to higher moderate-to-vigorous PA (MVPA) assessed by activity monitors at age 60–64 (Golubic et al., 2014). Similarly, in the Whitehall II study, self-reported PA frequency in midlife was associated with accelerometer-measured MVPA 13 years later (Hamer et al., 2012), and higher PA at age 40 recalled by 70–77 year-old Norwegians was related to higher accelerometer counts/minute (Viken et al., 2016).

It is unclear if greater PA across life cumulatively relates to greater volume of PA in later life, or if participation at later ages is more important. Moreover, few studies have objective measures of high impact PA in old age and none have examined the lifetime correlates of PA producing osteogenic vertical impacts at older age, including how different types of PA relate to higher impacts in later life. For example, walking is generally a lower impact activity that is unrelated to bone (Hannam et al., 2016a) whereas activities like jogging and dancing produce higher impacts, and participation in lower and higher impact PA could track across life. Therefore, in this study, we provide novel insights into determinants of high impact PA which may ultimately aid intervention design by using data from the Vertical Impacts on Bone in the Elderly (VIBE) study (Deere et al., 2016) to examine associations of walking and weight bearing exercise over the life course with accelerometer-measured high impact and overall PA in later life. We also examine the relative contributions of walking and weight bearing exercise to high impact PA at older age. We hypothesised that greater lifetime self-reported weight bearing exercise would be related to higher levels of osteogenic PA in later life and that greater participation in both walking and weight bearing exercise would translate to higher levels of high impacts.

## 2 Materials and methods

### 2.1 Study population

Participants were recruited to the VIBE study from the Cohort for Skeletal Health in Bristol and Avon (COSHIBA), Hertfordshire Cohort Study (HCS) and MRC NSHD. COSHIBA is a representative population-based cohort of 3200 women recruited through fifteen general practices in the Bristol and Avon area during 2007–2009, originally set up to investigate determinants of skeletal health in postmenopausal women (Clark et al., 2012). Only the 1286 COSHIBA participants who consented to be contacted about future research studies in 2014 and remained resident in the Bristol and Avon area were eligible to participate in the VIBE study. HCS comprises 3225 singleton births in Hertfordshire between 1931 and 1939 that still lived in the area during 1998–2003 (Syddall et al., 2005). Only the 443 HCS participants who were previously included in the UK arm of the European Project on Osteoarthritis (EPOSA) (Schaap et al., 2011) were invited to participate in VIBE. NSHD is a nationally representative sample of 5362 singleton births from one week in March 1946 (Kuh et al., 2011, Wadsworth et al., 2006). Most participants (79%) included in the home visit phase of the NSHD 24th data collection (2015–2016) (Kuh et al., 2016) were invited to participate in VIBE. In total, 3640 participants from the three cohorts were eligible to be invited to participate in the VIBE study.

Separate regional ethical approval was obtained for data collection in NSHD (14/LO/1073 and 14/SS/1009), HCS (10/HO311/59) and COSHIBA (14/SW/0138) and written informed consent was obtained from all participants.

### 2.2 Current PA

Participants who were invited and agreed to accelerometry monitoring, subject to availability of monitors, were provided with a GCDC X15-1c triaxial accelerometer (Gulf Coast Data Concepts, Waveland, Mississippi), custom designed size specific elasticated belt, a time log and a stamped addressed package along with written and, if seen in clinic or during a nurse home visit, verbal instructions. Accelerometers were configured with standardised settings prior to participant use with a sampling frequency of 50 Hz to detect brief high impacts, a deadband setting of 0.1 g (the threshold which must be exceeded before a recording is made) and a timeout setting of 10 s (a single sample every 10 s is forced even if the recording is < 0.1 g), to ensure monitors record continuously for seven days on a single battery charge. Participants were instructed to wear the accelerometer securely positioned over their right hip pointing toward the centre of their body for seven continuous days, removing only for sleeping, washing and swimming. A time log was provided for participants to record when the monitor was put on in the morning and taken off at night for each monitoring day and any reason why that day had not been reflective of their normal activity.

Raw triaxial accelerometry data were uploaded to a secure shared drive and read into Stata 13 (StataCorp, College Station, TX) for standardised cleaning and processing by the coordinating centre, described in detail elsewhere (Deere et al., 2016). Briefly, vertical (Y-axis) accelerations data were cleaned to remove movement artefacts and non-wear time. Activity data were normalised based on seven valid days (≥ 10 h recording time) of 14 h. Y-axis peaks were calculated based on accelerations higher than the preceding and subsequent reading and recorded within 14 pre-specified g bands. We defined high impact PA as vertical acceleration peaks ≥ 1.5 g, where g values represent g over and above 1 g from earth's gravitational force, and was previously validated in older adults attending an exercise class (Hannam et al., 2016c). The ≥ 1.5 g cut-point for higher impacts was selected as very few impacts were observed within higher g bands (Deere et al., 2016, Hannam et al., 2016a, Hannam et al., 2016b). In addition, we derived a measure of overall PA by summing the total number of low, medium and higher magnitude acceleration peaks from all (i.e. x, y, z) axes. As VIBE aimed to investigate health benefits of higher impact PA in older adults, high impact PA was not assessed by accelerometer in younger populations.

### 2.3 Lifetime walking and weight-bearing exercise

At time of accelerometry assessment, participants reported, via questionnaire, their walking and weight bearing exercise (i.e. self-reported lower and higher impact PA respectively) at four periods covering the life course: up to age 18, between 18–29, 30–49 and since age 50 years. These represent childhood and adolescence, and earlier, mid and later adulthood respectively and are similar to categories used in previous studies (Hirvensalo et al., 2000, Kozakai et al., 2012). For walking, participants were asked: considering 20 min of brisk walking is about 1 mile, how many miles did/do you usually walk each day? Possible responses at each age category were under 1 mile, 1 to 2 miles, 3 to 5 miles, or > 5 miles. For weight bearing exercise, participants were asked: how often did/do you take part in sports and leisure time exercise involving weight bearing activity? (e.g. running, racquet sports, football, rugby, hockey and dancing – not including walking, cycling or swimming), which are activities that have been shown to produce higher impacts (Hannam et al., 2016c, Hannam et al., 2016b). Possible responses were none, occasionally (once a month), frequently (once a week) or very frequently (more than once a week). In addition to using the full range of responses, and to facilitate subsequent analyses described below, responses for each age category were also dichotomised as ≤ once a month or ≥ once a week for weight bearing activity and as ≤ 2 miles or ≥ 3 miles for walking).

### 2.4 Hypothesised confounders

Age, sex, socioeconomic position (SEP) and self-rated health were selected as confounders based on previous literature and included in analyses (Bauman et al., 2012). SEP was based on reports of educational attainment by age 26 and the main occupation during working life (using the 1990 Standard Occupational Classification code). Self-rated health was ranked from very good to very poor in HCS and COSHIBA and from excellent to poor in NSHD.

### 2.5 Statistical analysis

Associations of reported miles walked and weight bearing exercise in each age category with monitored high impact and overall PA in later life were examined using linear regressions. We used interaction terms to test age, sex and cohort differences and subsequent analyses were performed on all participants combined after only two interactions were found (walking up to 18-high impact PA (*P* = 0.02) and weight bearing exercise up to 18-overall PA (*P* = 0.02)). Initial models were adjusted for age, sex and cohort and then for SEP and self-rated health. Final models were further adjusted for walking or weight bearing exercise in other age categories.

Responses at each age category were then summed to derive categorical lifetime walking and weight bearing exercise scores (range: 0 (least active) to 4 (most active)), and their associations with later life PA were examined using linear regression models with similar adjustment for confounders. To test whether this accumulation model which assumes similar effect sizes at each age, an alternative accumulation model that allows for different effect sizes at different ages or a sensitive periods model best fit the data, we used a structured approach (Mishra et al., 2009) to formally compare models (see Appendix A). This involved comparing nested models with a fully saturated model that assumes all possible trajectories of weight bearing activity/walking are related to later life PA. Large *P*-values indicate that the nested model fits the data as well as the saturated model and, therefore, is supported by the data (Mishra et al., 2009).

To examine the contribution of both walking and weight bearing exercise from age 50 to PA in later life, we grouped participants as 1) walking up to 2 miles and none/occasional weight bearing exercise, 2) walking at least 3 miles and none/occasional weight bearing exercise, 3) walking up to 2 miles and frequent/very frequent weight bearing exercise, or 4) walking at least 3 miles and frequent/very frequent weight bearing exercise. These models were adjusted for the above-mentioned confounders plus prior walking and weight bearing exercise. Finally, two sensitivity analyses were performed. First, we repeated analyses using the full range of responses for walking and weight bearing exercise at each age category. Second, we used prospective data on self-reported walking for pleasure and weight bearing exercise obtained via postal questionnaires from one of the participating VIBE cohorts; the MRC NSHD at ages 36 (based on any participation in the previous 4 weeks) and 60–64 years (any participation in the previous 12 months) and examined their relation to later life accelerometer-measured high impact and overall PA.

## 3 Results

### 3.1 Descriptive statistics

**Fig. 1:**
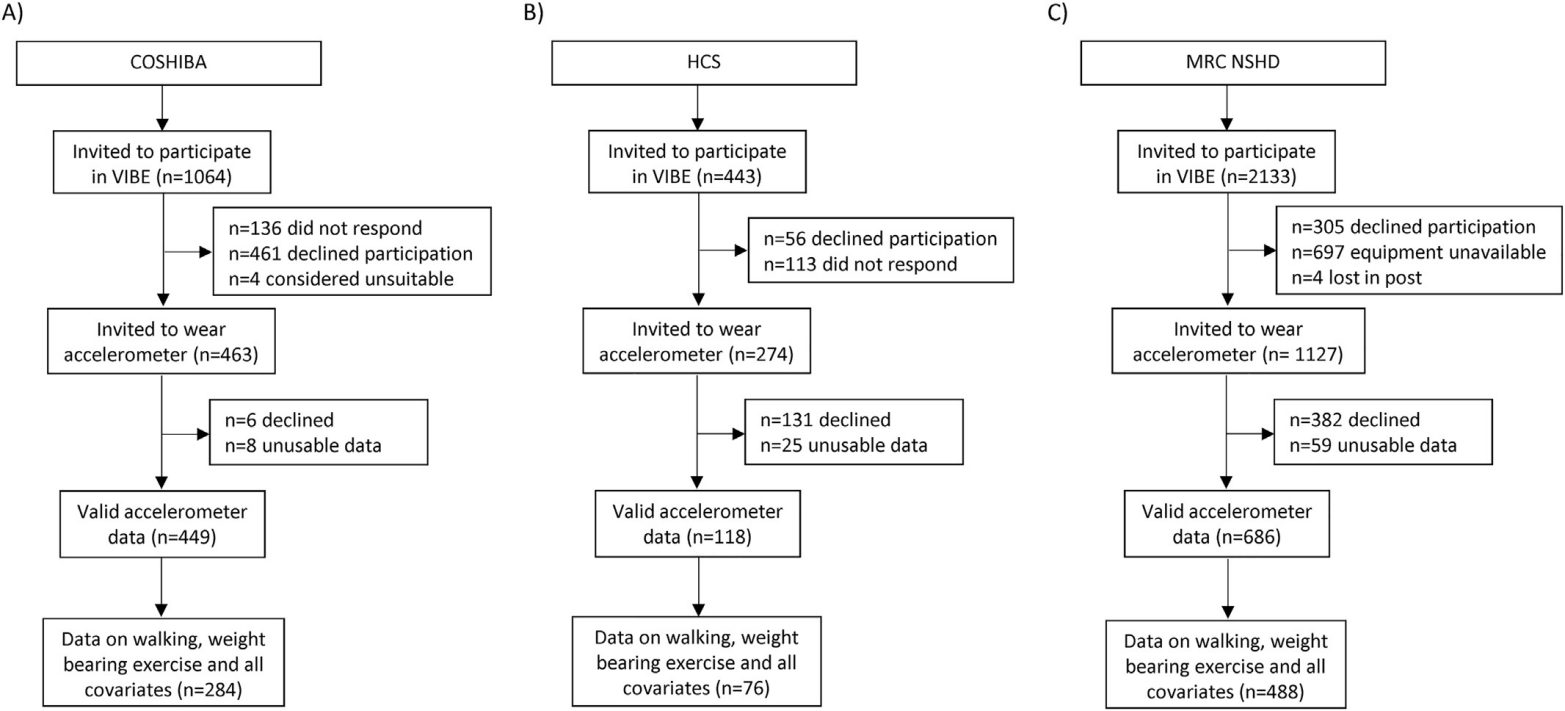
Study flowchart. Recruitment of VIBE study participants from A) COSHIBA, B) HCS and C) MRC NSHD. COSHIBA: Cohort for Skeletal Health in Bristol and Avon. HCS: Hertfordshire Cohort Study. MRC NSHD: Medical Research Council National Survey of Health and Development.

A total of 848 participants (72.8% female) aged between 69 and 88 years (mean = 72.4) were included in analyses (Fig. 1). Of these, 284 were from COSHIBA (100% female, mean age = 76.6), 488 from NSHD (50.2% female, mean age = 69) and 76 from HCS (42.1% female, mean age = 78.4) (Fig. 1). Men had higher accelerometer-measured overall PA and high impact PA than women. Men reported walking longer distances up to age 18 and since age 50 than women but there was little difference in miles walked between ages 18 and 49 (Table 1). Men reported more frequent weight bearing exercise at all ages up to age 50 but women indicated more frequent participation in weight bearing exercise since age 50 (Table 1). For both walking and weight bearing exercise, correlations were moderate and weakened as the time between ages increased, whereas at any age category, the two activity types (i.e. walking and weight bearing exercise) were only weakly correlated (r = 0.1 to 0.2) (Appendix B). Over a third of the sample (38%) had an A-level or higher educational qualification and more than half (53%) were in the highest two occupational classes. The majority rated their health as good, very good or excellent (86%).

### 3.2 Lifetime daily miles walked and later life PA

Longer daily walking distance since age 50 was similarly associated with greater overall and high impact PA in later life including after adjustment for confounders (Table 2). In contrast to other results, greater daily distances walked up to age 18 was associated with less later life PA and this was maintained after adjustment. When compared with the least lifetime walking, those reporting the most had higher overall, but not high impact, PA (Table 2). The structured approach suggested that a lifetime accumulation model allowing for differences in effect sizes at different ages provided the best fit to the data (overall PA: *P* = 0.3, high impact PA; *P* = 0.1) (see Appendix A).

### 3.3 Lifetime weight-bearing exercise and later life PA

More frequent weight bearing exercise at each age was related to higher later life PA in initial models however, after adjustment for confounders and previous/later weight bearing exercise, associations were only observed for the ‘since age 50’ period (Table 3). When compared with the least lifetime weight bearing exercise, those reporting the most had higher overall and high impact PA (Table 3). The structured approach suggested that a lifetime accumulation model allowing for differences in effect sizes provided the best fit to the data (overall PA: *P* = 0.8, high impact PA: *P* = 0.7). There was also evidence for a sensitive period (‘since age 50’) model for high impact PA (*P* = 0.8) (see Appendix A).

### 3.4 Walking and weight bearing exercise since age 50 and later life PA

**Fig. 2:**
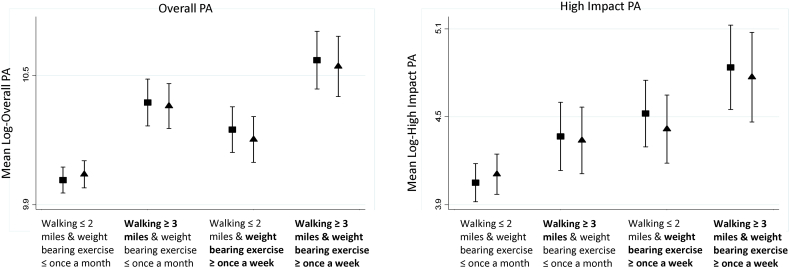
Daily miles walked and weight bearing exercise since age 50 in relation to accelerometer-measured overall and high impact physical activity (PA) in later life. N = 848 (≤ 2 miles walking and ≤ once a month weight bearing exercise (n = 457), ≥ 3 miles walking and ≤ once a month weight bearing exercise (n = 145), ≤ 2 miles walking and ≥ once a week weight bearing exercise (n = 153), ≥ 3 miles walking and ≥ once a week weight bearing exercise (n = 93)). Adjusted means calculated from linear regression. Model 1 (square): adjusted for age, sex, and cohort. Model 2 (triangle): further adjusted for education, occupational class, self-rated health, walking speed, mental wellbeing and all prior walking and weight bearing exercise. *P*-values from overall tests of association: (a) overall PA = model 1: *P* < 0.001, model 2: *P* < 0.001, (b) high impact PA = model 1: *P* < 0.001, model 2: *P* = 0.005.

When compared with walking up to 2 miles and none/occasional weight bearing exercise since age 50, those reporting greater distances walked and more frequent participation in weight bearing exercise had the highest overall and high impact PA in later life (Fig. 2). Those reporting similar walking but more frequent weight bearing exercise had higher levels of high impact PA whereas those reporting longer distances walked but similar amounts of weight bearing exercise had higher overall PA (Fig. 2). Associations were slightly attenuated after adjustment for confounders and walking and weight bearing exercise history (Fig. 2).

**Table 1:** Numbers (%) in each group of lifetime walking and weight bearing exercise by sex.

	Women (n = 617)	Men (n = 301)	*P* (sex difference)
Daily miles walked: up to 18 yr.			0.06
≤ 2 miles	303 (54.0)	135 (47.0)	
≥ 3 miles	258 (46.0)	152 (53.0)	
Daily miles walked: 18–29 yr.			0.7
≤ 2 miles	296 (52.8)	148 (51.6)	
≥ 3 miles	265 (47.2)	139 (48.4)	
Daily miles walked: 30–49 yr.			0.9
≤ 2 miles	338 (60.3)	171 (59.6)	
≥ 3 miles	223 (39.8)	116 (40.4)	
Daily miles walked: 50 + yr.			0.06
≤ 2 miles	415 (74.0)	195 (68.0)	
≥ 3 miles	146 (26.0)	92 (32.1)	
Lifetime daily miles walked			0.4
0 (≤ 2 miles at each age category)	198 (35.3)	85 (29.6)	
1	91 (16.2)	51 (17.8)	
2	94 (16.8)	54 (18.8)	
3	99 (17.7)	48 (16.7)	
4 (≥ 3 miles at each age category)	79 (14.1)	49 (17.1)	
Weight bearing exercise: up to 18 yr.			0.2
≤ once a month (none/occasionally)	169 (30.1)	74 (25.8)	
≥ once a week (frequently/very frequently)	392 (69.9)	213 (74.2)	
Weight bearing exercise: 18–29 yr.			< 0.001
≤ once a month (none/occasionally)	326 (58.1)	112 (39.0)	
≥ once a week (frequently/very frequently)	235 (41.9)	175 (61.0)	
Weight bearing exercise: 30–49 yr.			0.1
≤ once a month (none/occasionally)	343 (61.1)	159 (55.4)	
≥ once a week (frequently/very frequently)	218 (38.9)	128 (44.6)	
Weight bearing exercise: 50 + yr.			0.02
≤ once a month (none/occasionally)	383 (68.3)	219 (76.3)	
≥ once a week (frequently/very frequently)	178 (31.7)	68 (23.7)	
Lifetime weight-bearing exercise			0.02
0 (≤ once a month at each age category)	114 (20.3)	51 (17.8)	
1	149 (26.6)	55 (19.2)	
2	117 (20.9)	65 (22.7)	
3	84 (15.0)	65 (22.7)	
4 (≥ once a week at each age category)	97 (17.3)	51 (17.8)	
Walking & weight bearing exercise: 50 + yr.			0.009
≤ 2 miles walking & ≤ once a month weight bearing exercise	297 (52.9)	160 (55.8)	
≥ 3 miles walking & ≤ once a month weight bearing exercise	86 (15.3)	59 (20.6)	
≤ 2 miles walking & ≥ once a week weight bearing exercise	118 (21.0)	35 (12.2)	
≥ 3 miles walking & ≥ once a week weight bearing exercise	60 (10.7)	33 (11.5)	

N = 848: data on all accelerometer outcomes, age, sex, education, occupational class, self-rated health and lifetime walking and weight bearing exercise. ≤ once a month (0): None or once a month (occasionally). ≥ once a week (1): once a week (frequently) or more than once week (very frequently). ≤ 2 miles (0): under 1 mile or 1 to 2 miles. ≥ 3 miles (1): 3 to 5 miles or > 5 miles.

**Table 2:** Lifetime daily miles walked in relation to accelerometer-measured overall and high impact physical activity (PA) in later life.

	Log-overall PA				Log-high impact PA			
	Model 1 β (95% CI)	*P*	Model 2 β (95% CI)	*P*	Model 1 β (95% CI)	*P*	Model 2 β (95% CI)	*P*
Up to 18 yrs.								
≤ 2 miles (n = 438)	0.0	0.03	0.0	0.04	0.0	0.1	0.0	0.05
≥ 3 miles (n = 410)	− 0.106 (− 0.199, − 0.014)		− 0.112 (− 0.216, − 0.008)		− 0.152 (− 0.347, 0.043)		− 0.228 (− 0.458, 0.002)	
18–29 yrs.								
≤ 2 miles (n = 444)	0.0	0.1	0.0	0.02	0.0	> 0.9	0.0	0.9
≥ 3 miles (n = 404)	− 0.074 (− 0.167, 0.020)		− 0.147 (− 0.267, − 0.027)		− 0.006 (− 0.202, 0.190)		− 0.013 (− 0.279, 0.253)	
30–49 yrs.								
≤ 2 miles (n = 509)	0.0	0.008	0.0	0.2	0.0	0.08	0.0	0.3
≥ 3 miles (n = 309)	0.127 (0.033, 0.221)		0.085 (− 0.037, 0.206)		0.180 (− 0.019, 0.378)		0.134 (− 0.135, 0.402)	
50 + yrs.								
≤ 2 miles (n = 610)	0.0	< 0.001	0.0	< 0.001	0.0	< 0.001	0.0	0.006
≥ 3 miles (n = 238)	0.383 (0.282, 0.484)		0.383 (0.273, 0.494)		0.390 (0.173, 0.606)		0.344 (0.099, 0.589)	
Lifetime walking score								
0 (n = 283)	0.0	0.005	0.0	0.005	0.0	0.3	0.0	0.4
1 (n = 142)	0.156 (0.018, 0.294)		0.119 (− 0.012, 0.250)		0.205 (− 0.087, 0.496)		0.135 (− 0.148, 0.418)	
2 (n = 148)	0.022 (− 0.114, 0.159)		0.002 (− 0.127, 0.132)		0.235 (− 0.053, 0.522)		0.214 (− 0.066, 0.493)	
3 (n = 147)	0.015 (− 0.152, 0.122)		− 0.021 (− 0.151, 0.109)		0.037 (− 0.252, 0.325)		0.041 (− 0.240, 0.322)	
4 (n = 128)	0.228 (0.085, 0.372)		0.224 (0.087, 0.362)		0.226 (− 0.076, 0.528)		0.239 (− 0.058, 0.536)	

N = 848. Overall PA: overall acceleration vector magnitude (sum of low, medium and high magnitude acceleration peaks in X, Y and Z axes). High impact PA: vertical (Y) axis peaks measuring ≥ 1.5 g. ≤ 2 miles: under 1 mile or 1 to 2 miles. ≥ 3 miles: 3 to 5 miles or over 5 miles. Lifetime walking score: 0: ≤ 2 miles at each age period, 1: ≥ 3 miles at one age period, 2: ≥ 3 miles at two age periods, 3: ≥ 3 miles at three age periods, 4: ≥ 3 miles at each age period. Model 1: adjusted for age, sex, and cohort. Model 2: further adjusted for educational level, occupational class, self-rated health and miles walked at all previous and/or later age categories (except for the cumulative scores). *P*-values from overall tests of association.

**Table 3:** Lifetime weight bearing exercise in relation to accelerometer-measured overall and high impact physical activity (PA) in later life.

	Log-overall PA				Log-high impact PA			
	Model 1 β (95% CI)	*P*	Model 2 β (95% CI)	*P*	Model 1 β (95% CI)	*P*	Model 2 β (95% CI)	*P*
Up to 18 yrs.								
≤ once a month (n = 243)	0.0	0.001	0.0	0.05	0.0	0.02	0.0	0.5
≥ once a week (n = 605)	0.182 (0.080, 0.285)		0.115 (0.002, 0.227)		0.262 (0.046, 0.478)		0.079 (− 0.163, 0.322)	
18–29 yrs.								
≤ once a month (n = 438)	0.0	0.04	0.0	0.7	0.0	0.05	0.0	0.8
≥ once a week (n = 410)	0.102 (0.006, 0.199)		− 0.022 (− 0.138, 0.094)		0.201 (0.000, 0.403)		0.031 (− 0.218, 0.280)	
30–49 yrs.								
≤ once a month (n = 502)	0.0	< 0.001	0.0	0.4	0.0	0.001	0.0	0.6
≥ once a week (n = 346)	0.179 (0.085, 0.273)		0.052 (− 0.066, 0.170)		0.330 (0.132, 0.528)		0.073 (− 0.180, 0.327)	
50 + yrs.								
≤ once a month (n = 602)	0.0	< 0.001	0.0	0.001	0.0	< 0.001	0.0	0.002
≥ once a week (n = 246)	0.276 (0.174, 0.378)		0.193 (0.078, 0.308)		0.521 (0.307, 0.735)		0.386 (0.138, 0.634)	
Lifetime weight bearing exercise score								
0 (n = 165)	0.0	< 0.001	0.0	< 0.001	0.0	< 0.001	0.0	0.002
1 (n = 204)	0.188 (0.049, 0.328)		0.099 (− 0.037, 0.235)		0.261 (− 0.033, 0.554)		0.076 (− 0.216, 0.369)	
2 (n = 182)	0.138 (− 0.006, 0.282)		0.087 (− 0.053, 0.226)		0.183 (− 0.121, 0.486)		0.080 (− 0.221, 0.380)	
3 (n = 149)	0.217 (0.066, 0.368)		0.161 (0.015, 0.307)		0.348 (0.029, 0.667)		0.222 (− 0.092, 0.537)	
4 (n = 148)	0.434 (0.281, 0.587)		0.348 (0.201, 0.495)		0.754 (0.432, 1.076)		0.587 (0.270, 0.904)	

N = 848. Overall PA: overall acceleration vector magnitude (sum of low, medium and high magnitude acceleration peaks in X, Y and Z axes). High impact PA: vertical (Y) axis peaks ≥ 1.5 g. ≤ once a month: none or once a month (occasionally). ≥ once a week: once a week (frequently) or more than once week (very frequently). Lifetime weight bearing exercise score: 0: ≤ once a month at each age period, 1: ≥ once a week at one age period, 2: ≥ once a week at two age periods, 3: ≥ once a week at three age periods, 4: ≥ once a week at each age period. Model 1: adjusted for age, sex, and cohort. Model 2: further adjusted for educational level, occupational class, self-rated health and weight-bearing exercise at all previous and/or later age categories (except for the cumulative scores). *P*-values from overall tests of association.

### 3.5 Sensitivity analyses

Repeating analyses using the full range of responses at each age category led to similar findings (see Appendix C). Broadly similar results were found when examining prospectively-reported walking and weight bearing exercise at age 36 and 60–64 (which weakly correlated with the retrospective reports) in relation to later PA in NSHD though overall, estimates were less precise, likely due to the smaller sample size (see Appendix D).

## 4 Discussion

We examined how patterns of self-reported walking and leisure-time weight bearing exercise over the life course relate to accelerometer-measured high impact PA and overall PA in older Britons from three population-based cohorts. Greater distances walked throughout life was related to higher overall, but not high impact, PA whereas more frequent weight bearing exercise over the life course was related to both higher overall PA and high impact PA. Associations were strongest from midlife and independent of selected confounders. Those reporting the most walking and weight bearing exercise from midlife had highest levels of subsequent overall and high impact PA.

Consistent with our findings of stronger associations with activities from age 50, a study of older Japanese who recalled lifetime exercise (regular PA and sports for at least 20 min, once/week and over 1 year) found that exercise in the most recent age category (40–59 years) was the strongest predictor of current self-reported exercise levels (Kozakai et al., 2012). Likewise, a Swedish study of recalled lifetime overall PA (Frandin et al., 1995) and a Finnish study of past competitive and recreational sports (Hirvensalo et al., 2000) both found that the latest age categories (66–76 and 40–64 years respectively) were most strongly related to current self-reported PA. Our findings are also consistent with a German study showing participants aged > 50 who recalled greater numbers of sports years had lower likelihood of dropping out of sport (Engel and Nagel, 2011). Our study is important because it shows that lifetime walking and weight bearing exercise are related to accelerometer-measured high impact PA at old age.

That weight bearing exercise (in sports and during leisure-time) appeared more strongly related to high impact PA than walking is consistent with tracking of higher impact activities across life (Telama, 2009). This tracking may also reflect habits and behavioural processes, and it could be that maintenance of function and lower debilitating illness because of prior PA facilitates maintenance of PA at older age (Fox et al., 2015). This finding also suggests that previously reported beneficial associations between lifetime weight bearing exercise and bone in older adults (Daly and Bass, 2006) may be due to osteogenic higher impact PA in later life (Hannam et al., 2016a, Martyn-St James and Carroll, 2009, Tobias et al., 2014). We found that those reporting the highest levels of both walking and weight bearing exercise since age 50 had the highest levels of later life PA, which is consistent with findings that 30–80-year-old Finns participating in more than one type of PA had highest levels of self-reported PA 22 years later (Borodulin et al., 2012). That higher walking up to age 18 was inversely related to later life PA might be explained by people walking greater distances out of necessity (e.g. due to lack of resources) at earlier ages whereas at later ages people might walk greater distances through choice including as an active leisure pursuit.

Strengths of this study include use of raw accelerometer data to derive novel measures of high impact PA and overall PA, including participants from three population-based cohorts comprising a broad age range of older adults which helps increase power and generalisability, and cross-evaluation of walking and weight bearing exercise history analyses with prospectively-reported measures. Lifetime walking and exercise were retrospectively reported and thus recall errors (and reporting bias) are possible, particularly long-term recall and especially as participants were aged 69 and older (Schrack et al., 2016). Consequently, the closer temporality between the latest age category and accelerometer assessments may explain why associations with overall and high impact PA were strongest for the ‘since age 50’ category. Nevertheless, sensitivity analyses using prospectively reported walking and weight bearing exercise across adulthood from NSHD participants led to comparable findings.

Further, despite their strengths, accelerometers are currently unable to provide contextual information on PA and so it was not possible to identify which specific types of PA were producing higher impacts. Our findings may also be susceptible to survival bias which would underestimate associations as those healthiest and most active survive to old age, possibly because of lower debilitating illness and greater maintenance of physical function allowing continued participation in PA. Likewise, our findings may have been influenced by inclusion of participants who are more aware of the benefits of PA and keep a more active lifestyle than the general population, particularly as VIBE participants tended to have lower BMI and higher educational level compared with other participants from the three contributing studies who did not participate in VIBE (Hannam et al., 2016b).

Our findings suggest that continued participation in both walking and weight bearing exercise may be important for both overall and higher impact PA in older age. To this end, supporting both walking and weight bearing exercises, including activities like tennis and dancing (Hannam et al., 2016b), from midlife may be important for an active and functional older life including better skeletal health by promoting both overall and higher impact PA in later life. In addition, aerobics classes may provide a safe way for older adults to achieve exposure to higher impacts (Hannam et al., 2016c). In conclusion, we found that cumulative exposure to walking and weight bearing exercise across life was related to greater levels of higher impact PA in later life, and that associations were strongest from midlife.

The following are the supplementary data related to this article.

Appendix A Life course models used. Appendix A

Appendix B Spearman rank correlation coefficients for lifetime daily miles walked and weight bearing exercise. Appendix B

Appendix C Lifetime self-reported walking and weight-bearing exercise in relation to accelerometer-measured overall and high impact physical activity (PA) in later life. Appendix C

Appendix D Prospectively reported walking and weight bearing exercise at ages 36 and 60–64 in relation to accelerometer-measured overall and high impact physical activity (PA) at age 69 in the MRC NSHD. Appendix D
