# Supplementary material for: Associations of lifetime walking and weight bearing exercise with accelerometer-measured high impact physical activity in later life
Source: Prev Med Rep. 2017 Oct 25;8:183–9. doi: 10.1016/j.pmedr.2017.10.011 (PMC5671612; doi:10.1016/j.pmedr.2017.10.011)
Supplement: Appendix A — Life course models used. [file mmc1.docx]

**Appendix A** Life course models used

Fully saturated model:

E(Y) = α + β_1_P_1_ + β_2_P_2_ + β_3_P_3_ + β_4_P_4_

+ θ_12_P_1_P_2_ + θ_13_P_1_P_3_ + θ_14_P_1_P_4_ + θ_23_P_2_P_3_ + θ_24_P_2_P_4_ + θ_34_P_3_P_4_ +

+ θ_123_P_1_P_2_P_3_ + θ_124_P_1_P_2_P_4_ + θ_134_P_1_P_3_P_4_ + θ_234_P_2_P_3_P_4_ +

+ θ_1234_P_1_P_2_P_3_P_4_

P_1_..P_4_ represent the walking and weight-bearing exercise responses from each of the four age categories (up to 18, 18-29, 30-49 and 50+ years).

|  | Daily miles walked | | Weight bearing exercise | |
| --- | --- | --- | --- | --- |
| Hypothesis and corresponding parameter constraints on regression analysis | Overall PA^a^ | High Impact PA^a^ | Overall PA^a^ | High Impact PA^a^ |
| Accumulation – effect size constrained to be the same at each time point  β_1_=β_2_=β_3_=β_4_  and  θ_12_=θ_13_=θ_14_=θ_23_=θ_24_=θ_34_=θ_123_=θ_124_=θ_134_=θ_234_=θ_1234_=0 | <0.001 | <0.001 | 0.1 | 0.06 |
| Accumulation - effect size allowed to vary between time points  β_1_≠β_2_≠β_3_≠β_4_  and  θ_12_=θ_13_=θ_14_=θ_23_=θ_24_=θ_34_=θ_123_=θ_124_=θ_134_=θ_234_=θ_1234_=0 | 0.3 | 0.1 | 0.8 | 0.7 |
| Sensitive period at up to 18 Yrs.  β_2_=β_3_=β_4_=θ_12_=θ_13_=θ_14_=θ_23_=θ_24_=θ_34_=θ_123_=θ_124_=θ_134_=θ_234_=θ_1234_=0 | <0.001 | <0.001 | 0.003 | <0.001 |
| Sensitive period at 18-29 Yrs.  β_1_=β_3_=β_4=_θ_12_=θ_13_=θ_14_=θ_23_=θ_24_=θ_34_=θ_123_=θ_124_=θ_134_=θ_234_=θ_1234_=0 | <0.001 | <0.001 | <0.001 | <0.001 |
| Sensitive period at 30-49 Yrs.  β_1_=β_2_=β_4=_θ_12_=θ_13_=θ_14_=θ_23_=θ_24_=θ_34_=θ_123_=θ_124_=θ_134_=θ_234_=θ_1234_=0 | <0.001 | <0.001 | 0.009 | 0.007 |
| Sensitive period at 50+ Yrs.  β_1_=β_2_=β_3=_θ_12_=θ_13_=θ_14_=θ_23_=θ_24_=θ_34_=θ_123_=θ_124_=θ_134_=θ_234_=θ_1234_=0 | <0.001 | 0.004 | 0.4 | 0.8 |

All life course models adjusted for age, sex and cohort.

^a^*P*-values from partial F-tests. Higher *P*-values indicate that the life course model shown fits the data as well as the fully saturated model.
