# Supplementary material for: Associations of lifetime walking and weight bearing exercise with accelerometer-measured high impact physical activity in later life
Source: Prev Med Rep. 2017 Oct 25;8:183–9. doi: 10.1016/j.pmedr.2017.10.011 (PMC5671612; doi:10.1016/j.pmedr.2017.10.011)
Supplement: Appendix B — Spearman rank correlation coefficients for lifetime daily miles walked and weight bearing exercise. [file mmc2.docx]

**Appendix B** Spearman rank correlation coefficients for lifetime daily miles walked and weight bearing exercise.

|  | Walking: up to 18 yr. | Walking: 18 to 29 yr. | Walking: 30 to 49 yr. | Walking: 50+ yr. | Weight bearing exercise: up to 18 yr. | Weight bearing exercise: 18 to 29 yr. | Weight bearing exercise: 30 to 49 yr. | Weight bearing exercise: 50+ yr. |
| --- | --- | --- | --- | --- | --- | --- | --- | --- |
| Walking: up to 18 yr. | 1.00 |  |  |  |  |  |  |  |
| Walking: 18 to 29 yr. | 0.6 | 1.00 |  |  |  |  |  |  |
| Walking: 30 to 49 yr. | 0.4 | 0.6 | 1.00 |  |  |  |  |  |
| Walking: 50+ yr. | 0.2 | 0.3 | 0.5 | 1.00 |  |  |  |  |
|  |  |  |  |  |  |  |  |  |
| Weight bearing exercise: up to 18 yr. | 0.1 | 0.03 | 0.00 | -0.03 | 1.00 |  |  |  |
| Weight bearing exercise: 18 to 29 yr. | 0.1 | 0.1 | 0.09 | 0.07 | 0.5 | 1.00 |  |  |
| Weight bearing exercise: 30 to 49 yr. | 0.1 | 0.1 | 0.1 | 0.1 | 0.3 | 0.5 | 1.00 |  |
| Weight bearing exercise: 50+ yr. | -0.02 | 0.04 | 0.08 | 0.1 | 0.1 | 0.3 | 0.5 | 1.00 |
