# Supplementary material for: Associations of lifetime walking and weight bearing exercise with accelerometer-measured high impact physical activity in later life
Source: Prev Med Rep. 2017 Oct 25;8:183–9. doi: 10.1016/j.pmedr.2017.10.011 (PMC5671612; doi:10.1016/j.pmedr.2017.10.011)
Supplement: Appendix C — Lifetime self-reported walking and weight-bearing exercise in relation to accelerometer-measured overall and high impact physical activity (PA) in later life. [file mmc3.docx]

**Appendix C1** Lifetime daily miles walked in relation to accelerometer-measured overall and high impact physical activity (PA) in later life.

|  | Log-Overall PA | | | |  | Log-High impact PA | | | |
| --- | --- | --- | --- | --- | --- | --- | --- | --- | --- |
|  | Model 1  β (95%CI) | p-value | Model 2  β (95%CI) | p-value |  | Model 1  β (95%CI) | p-value | Model 2  β (95%CI) | p-value |
|  |  |  |  |  |  |  |  |  |  |
| Up to 18 Yrs. |  |  |  |  |  |  |  |  |  |
| Under 1 mile (n=85) | 0.0 | 0.06 | 0.0 | 0.04 |  | 0.0 | 0.2 | 0.0 | 0.05 |
| 1 to 2 miles (n=353) | -0.004 (-0.167, 0.160) |  | -0.077 (-0.239, 0.084) |  |  | -0.026 (-0.370, 0.317) |  | -0.268 (-0.636, 0.101) |  |
| 3 to 5 miles (n=287) | -0.075 (-0.242, 0.092) |  | -0.183 (-0.363, -0.002) |  |  | -0.111 (-0.461, 0.240) |  | -0.485 (-0.895, -0.075) |  |
| ≥5 miles (n=123) | -0.191 (-0.382, 0.000) |  | -0.275 (-0.502, -0.048) |  |  | -0.322 (-0.723, 0.080) |  | -0.649 (-1.167, -0.132) |  |
|  |  |  |  |  |  |  |  |  |  |
| 18-29 Yrs. |  |  |  |  |  |  |  |  |  |
| Under 1 mile (n=96) | 0.0 | 0.2 | 0.0 | 0.09 |  | 0.0 | 0.2 | 0.0 | 0.3 |
| 1 to 2 miles (n=348) | 0.084 (-0.072, 0.240) |  | 0.136 (-0.034, 0.306) |  |  | 0.230 (-0.096, 0.556) |  | 0.293 (-0.094, 0.680) |  |
| 3 to 5 miles (n=295) | 0.016-0.143, 0.175) |  | -0.012 (-0.186, 0.211) |  |  | 0.244 (-0.090, 0.577) |  | 0.290 (-0.162, 0.742) |  |
| ≥5 miles (n=109) | -0.073 (-0.263, 0.116) |  | -0.025 (-0.292, 0.241) |  |  | -0.014 (-0.411, 0.383) |  | 0.017 (-0.590, 0.624) |  |
|  |  |  |  |  |  |  |  |  |  |
| 30-49 Yrs. |  |  |  |  |  |  |  |  |  |
| Under 1 mile (n=129) | 0.0 | 0.01 | 0.0 | 0.03 |  | 0.0 | 0.1 | 0.0 | 0.8 |
| 1 to 2 miles (n=380) | 0.059 (-0.079, 0.196) |  | -0.153 (-0.303, -0.002) |  |  | 0.243 (-0.047, 0.532) |  | 0.013 (-0.330, 0.356) |  |
| 3 to 5 miles (n=256) | 0.209 (0.064, 0.3550 |  | -0.080 (-0.266, 0.106) |  |  | 0.362 (0.056, 0.668) |  | 0.116 (-0.307, 0.539) |  |
| ≥5 miles (n=83) | 0.054 (-0.135, 0.244) |  | -0.273 (-0.528, 0.018) |  |  | 0.358 (-0.041, 0.756) |  | 0.250 (-0.331, 0.832) |  |
|  |  |  |  |  |  |  |  |  |  |
| 50+ Yrs. |  |  |  |  |  |  |  |  |  |
| Under 1 mile (n=221) | 0.0 | <0.001 | 0.0 | <0.001 |  | 0.0 | <0.001 | 0.0 | <0.001 |
| 1 to 2 miles (n=389) | 0.399 (0.291, 0.506) |  | 0.412 (0.300, 0.524) |  |  | 0.502 (0.266, 0.738) |  | 0.403 (0.147, 0.658) |  |
| 3 to 5 miles (n=186) | 0.612 (0.485, 0.739) |  | 0.686 (0.544, 0.828) |  |  | 0.661 (0.382, 0.940) |  | 0.602 (0.279, 0.926) |  |
| ≥5 miles (n=52) | 0.733 (0.536, 0.930) |  | 0.900 (0.685, 1.116) |  |  | 0.891 (0.499, 1.322) |  | 0.852 (0.362, 1.342) |  |

N=848. Overall PA: overall acceleration vector magnitude (sum of low, medium and high magnitude acceleration peaks in X, Y and Z axes). High impact PA: vertical (Y) axis peaks measuring ≥1.5g. Model 1: adjusted for age, sex, and cohort. Model 2: further adjusted for educational level, occupational class, self-rated health and miles walked at all previous and/or later age categories

**Appendix C2** Lifetime weight bearing exercise in relation to accelerometer-measured overall and high impact physical activity (PA) in later life

|  | Log-Overall PA | | | |  | Log-High impact PA | | | |
| --- | --- | --- | --- | --- | --- | --- | --- | --- | --- |
|  | Model 1  β (95%CI) | p-value | Model 2  β (95%CI) | p-value |  | Model 1  β (95%CI) | p-value | Model 2  β (95%CI) | p-value |
|  |  |  |  |  |  |  |  |  |  |
| Up to 18 Yrs. |  |  |  |  |  |  |  |  |  |
| None (n=145) | 0.0 | <0.001 | 0.0 | 0.008 |  | 0.0 | 0.1 | 0.0 | 0.5 |
| once a month (n=98) | 0.241 (0.065, 0.416) |  | 0.189 (0.011, 0.367) |  |  | -0.066 (-0.436, 0.305) |  | -0.224 (-0.608, 0.160) |  |
| once a week (n=281) | 0.302 (0.165, 0.440) |  | 0.242 (0.090, 0.394) |  |  | 0.213 (-0.078, 0.503) |  | -0.012 (-0.340, 0.315) |  |
| > once week (n=324) | 0.260 (0.125, 0.396) |  | 0.129 (-0.037, 0.294) |  |  | 0.255 (-0.031, 0.541) |  | -0.112 (-0.469, 0.246) |  |
|  |  |  |  |  |  |  |  |  |  |
| 18-29 Yrs. |  |  |  |  |  |  |  |  |  |
| None (n=240) | 0.0 | 0.01 | 0.0 | 0.2 |  | 0.0 | 0.03 | 0.0 | 0.5 |
| once a month (n=198) | 0.095 (-0.034, 0.225) |  | -0.031 (-0.180, 0.117) |  |  | 0.146 (-0.126, 0.418) |  | 0.064 (-0.255, 0.384) |  |
| once a week (n=245) | 0.093 (-0.032, 0.217) |  | -0.044 (-0.203, 0.116) |  |  | 0.157 (-0.105, 0.418) |  | 0.032 (-0.312, 0.376) |  |
| > once week (n=165) | 0.235 (0.093, 0.376) |  | 0.115 (-0.086, 0.316) |  |  | 0.452 (0.155, 0.749) |  | 0.274 (-0.158, 0.706) |  |
|  |  |  |  |  |  |  |  |  |  |
| 30-49 Yrs. |  |  |  |  |  |  |  |  |  |
| None (n=324) | 0.0 | 0.002 | 0.0 | 0.7 |  | 0.0 | 0.002 | 0.0 | >0.9 |
| once a month (n=178) | 0.036 (-0.090, 0.162) |  | 0.005 (-0.137, 0.148) |  |  | 0.110 (-0.155, 0.374) |  | 0.048 (-0.260, 0.356) |  |
| once a week (n=218) | 0.166 (0.048, 0.284) |  | 0.059 (-0.092, 0.210) |  |  | 0.264 (0.016, 0.512) |  | 0.056 (-0.269, 0.381) |  |
| > once week (n=128) | 0.238 (0.096, 0.380) |  | -0.022 (-0.224, 0.180) |  |  | 0.553 (0.255, 0.851) |  | -0.031 (-0.466, 0.405) |  |
|  |  |  |  |  |  |  |  |  |  |
| 50+ Yrs. |  |  |  |  |  |  |  |  |  |
| None (n=461) | 0.0 | <0.001 | 0.0 | 0.01 |  | 0.0 | <0.001 | 0.0 | 0.003 |
| once a month (n=141) | 0.002 (-0.127, 0.131) |  | -0.039 (-0.178, 0.101) |  |  | 0.074 (-0.196, 0.344) |  | 0.031 (-0.270, 0.333) |  |
| once a week (n=145) | 0.245 (0.116, 0.374) |  | 0.152 (0.008, 0.296) |  |  | 0.322 (0.053, 0.592) |  | 0.224 (-0.086, 0.535) |  |
| > once week (n=101) | 0.320 (0.173, 0.467) |  | 0.222 (0.047, 0.397) |  |  | 0.847 (0.539, 1.154) |  | 0.693 (0.316, 1.070) |  |

N=848. Overall PA: overall acceleration vector magnitude (sum of low, medium and high magnitude acceleration peaks in X, Y and Z axes). High impact PA: vertical (Y) axis peaks measuring ≥1.5g. Model 1: adjusted for age, sex, and cohort. Model 2: further adjusted for educational level, occupational class, self-rated health and all prior weight-bearing PA at all previous and/or later age categories
