# Supplementary material for: Associations of lifetime walking and weight bearing exercise with accelerometer-measured high impact physical activity in later life
Source: Prev Med Rep. 2017 Oct 25;8:183–9. doi: 10.1016/j.pmedr.2017.10.011 (PMC5671612; doi:10.1016/j.pmedr.2017.10.011)
Supplement: Appendix D — Prospectively reported walking and weight bearing exercise at ages 36 and 60–64 in relation to accelerometer-measured overall and high impact physical activity (PA) at age 69 in the MRC NSHD. [file mmc4.docx]

**Appendix D** Prospectively reported walking and weight bearing exercise at ages 36 and 60-64 in relation to accelerometer-measured overall and high impact physical activity (PA) at age 69 in the MRC NSHD.

|  | Log-Overall PA | | | |  | Log-High impact PA | | | |
| --- | --- | --- | --- | --- | --- | --- | --- | --- | --- |
|  | Model 1  β (95%CI) | p-value | Model 2  β (95%CI) | p-value |  | Model 1  β (95%CI) | p-value | Model 2  β (95%CI) | p-value |
|  |  |  |  |  |  |  |  |  |  |
| Leisure walking |  |  |  |  |  |  |  |  |  |
| Age 36 |  |  |  |  |  |  |  |  |  |
| No (n=130) | 0.0 | 0.01 | 0.0 | 0.2 |  | 0.0 | 0.06 | 0.0 | 0.4 |
| Yes (n=352) | 0.166 (0.036, 0.296) |  | 0.077 (-0.050, 0.204) |  |  | 0.288 (-0.015, 0.590) |  | 0.125 (-0.177, 0.427) |  |
|  |  |  |  |  |  |  |  |  |  |
| Age 60-64 |  |  |  |  |  |  |  |  |  |
| No (n=92) | 0.0 | <0.001 | 0.0 | 0.06 |  | 0.0 | 0.02 | 0.0 | 0.4 |
| Yes (n=390) | 0.263 (0.118, 0.409) |  | 0.140 (-0.005, 0.285) |  |  | 0.407 (0.065, 0.748) |  | 0.148 (-0.200, 0.496) |  |
|  |  |  |  |  |  |  |  |  |  |
| Weight bearing exercise |  |  |  |  |  |  |  |  |  |
| Age 36 |  |  |  |  |  |  |  |  |  |
| No (n=277) | 0.0 | 0.05 | 0.0 | 0.1 |  | 0.0 | 0.2 | 0.0 | 0.3 |
| Yes (n=205) | 0.118 (-0.001, 0.237) |  | 0.096 (-0.018, 0.209) |  |  | 0.190 (-0.083, 0.464) |  | 0.136 (-0.131, 0.404) |  |
|  |  |  |  |  |  |  |  |  |  |
| Age 60-64 |  |  |  |  |  |  |  |  |  |
| No (n=246) | 0.0 | 0.007 | 0.0 | 0.3 |  | 0.0 | 0.002 | 0.0 | 0.08 |
| Yes (n=236) | 0.164 (0.046, 0.282) |  | 0.061 (-0.053, 0.176) |  |  | 0.421 (0.151, 0.691) |  | 0.242 (-0.027, 0.511) |  |
|  |  |  |  |  |  |  |  |  |  |
| Walking and weight bearing exercise at age 60-64 |  |  |  |  |  |  |  |  |  |
| None (n=72) | 0.0 | 0.002 | 0.0 | 0.2 |  | 0.0 | 0.005 | 0.0 | 0.2 |
| Walking only (n=173) | 0.134 (-0.041, 0.310) |  | 0.037 (-0.136, 0.210) |  |  | 0.200 (-0.210, 0.610) |  | -0.005 (-0.418, 0.409) |  |
| Weight bearing exercise only (n=19) | -0.033 (-0.356, 0.291) |  | -0.125 (-0.434, 0.184) |  |  | 0.210 (-0.544, 0.964) |  | 0.025 (-0.714, 0.764) |  |
| Walking and weight bearing exercise (n=214) | 0.294 (0.122, 0.466) |  | 0.120 (-0.054, 0.294) |  |  | 0.623 (0.222, 1.023) |  | 0.284 (-0.132, 0.700) |  |

Overall PA: overall acceleration vector magnitude (sum of low, medium and high magnitude acceleration peaks in X, Y and Z axes). High impact PA: vertical (Y) axis peaks measuring ≥1.5g. Model 1: adjusted for sex. Model 2: further adjusted for educational level, occupational class, self-rated health and walking and/or weight-bearing exercise at age 36/ 60-64. Wight bearing exercise includes netball, volleyball, basketball, bowling, cricket, Football/rugby/hockey, squash, tennis, badminton, table tennis, dancing, backpacking, hill walking, mountain climbing, running, jogging, high impact aerobics, other aerobics, martial arts and weight training.
